# Standardization of the Agar Plate Method for Bacteriophage Production

**DOI:** 10.3390/antibiotics14010002

**Published:** 2024-12-24

**Authors:** Su Jin Jo, Young Min Lee, Kevin Cho, Seon Young Park, Hyemin Kwon, Sib Sankar Giri, Sung Bin Lee, Won Joon Jung, Jae Hong Park, Mae Hyun Hwang, Da Sol Park, Eun Jae Park, Sang Wha Kim, Jin Woo Jun, Sang Guen Kim, Ji Hyung Kim, Se Chang Park

**Affiliations:** 1 Laboratory of Aquatic Biomedicine, College of Veterinary Medicine and Research Institute for Veterinary Science, Seoul National University, Seoul 08826, Republic of Korea; ssjjone@snu.ac.kr (S.J.J.);; 2 Division of Animal and Dairy Sciences, College of Agriculture and Life Science, Chungnam National University, Daejeon 34134, Republic of Korea; 3 Department of Microbiology and Molecular Biology, College of Bioscience and Biotechnology, Chungnam National University, Daejeon 34134, Republic of Korea; 4 College of Veterinary Medicine & Institute of Veterinary Science, Kangwon National University, Chuncheon 24341, Republic of Korea; 5 Department of Aquaculture, Korea National College of Agriculture and Fisheries, Jeonju 54874, Republic of Korea; 6 Laboratory of Phage and Microbial Resistance, Department of Biological Sciences, Kyonggi University, Suwon 16227, Republic of Korea; 7 Department of Food Science and Biotechnology, College of Bionano Technology, Gachon University, Seongnam 13120, Republic of Korea

**Keywords:** bacteriophage, agar plate method, standardization, personalize therapy

## Abstract

The growing threat of antimicrobial resistance (AMR), exacerbated by the COVID-19 pandemic, highlights the urgent need for alternative treatments such as bacteriophage (phage) therapy. Phage therapy offers a targeted approach to combat bacterial infections, particularly those resistant to conventional antibiotics. This study aimed to standardize an agar plate method for high-mix, low-volume phage production, suitable for personalized phage therapy. Plaque assays were conducted with the double-layer agar method, and plaque sizes were precisely measured using image analysis tools. Regression models developed with Minitab software established correlations between plaque size and phage production, optimizing production while minimizing resistance development. The resulting Plaque Size Calculation (PSC) model accurately correlated plaque size with inoculum concentration and phage yield, establishing specific plaque-forming unit (PFU) thresholds for optimal production. Using phages targeting pathogens such as *Escherichia*, *Salmonella*, *Staphylococcus*, *Pseudomonas*, *Chryseobacterium*, *Vibrio*, *Erwinia*, and *Aeromonas* confirmed the model’s accuracy across various conditions. The model’s validation showed a strong inverse correlation between plaque size and minimum-lawn cell clearing PFUs (MCPs; R² = 98.91%) and identified an optimal inoculum density that maximizes yield while minimizing the evolution of resistant mutants. These results highlight that the PSC model offers a standardized and scalable method for efficient phage production, which is crucial for personalized therapy and AMR management. Furthermore, its adaptability across different conditions and phages positions it as a potential standard tool for rapid and precise phage screening and propagation in both clinical and industrial settings.

## 1. Introduction

The growing threat of antimicrobial resistance (AMR) presents a significant global health challenge, with recent estimates indicating that AMR-related infections could lead to millions of deaths annually by 2050 if left unaddressed [1,2,3,4]. The rise of AMR is largely driven by the overuse of antibiotics and the slow pace of new antimicrobial agent development, creating an urgent need for innovative therapeutic approaches [5,6]. In addition to its health impacts, AMR poses a substantial economic burden, increasing healthcare costs and leading to productivity losses worldwide [7,8]. A key challenge in addressing AMR lies in its ability to render conventional antibiotics ineffective, significantly limiting treatment options for life-threatening infections [9,10].

Bacteriophages (phages) are emerging as promising alternatives to antibiotics, largely due to their unique ability to specifically target and replicate within bacterial cells [11,12]. Unlike antibiotics, phage therapy introduces virulent phages that selectively infect and lyse clinically relevant bacterial pathogens without disrupting the normal microbiome [13,14,15]. Phages multiply at infection sites through a dynamic lytic cycle, ensuring localized bacterial elimination [16,17]. These unique characteristics make phage therapy particularly suited to combat antibiotic-resistant bacterial strains, which are increasingly difficult to treat using conventional approaches [18,19]. Efforts to broaden phage therapy’s accessibility include clinical trials across Europe and the United States, demonstrating phages’ potential to combat antimicrobial-resistant bacteria [20,21]. These efforts have also extended to the food and agriculture sectors, where phages are being marketed for preventing foodborne infections and managing plant bacterial diseases [22,23,24,25].

As demand for phages in industries like agriculture and aquaculture grows, large-scale production methods, particularly broth cultures, have become golden standard [26,27]. Production systems such as batch and continuous cultures are commonly optimized to increase yields, adjusting factors like host cell density, multiplicity of infection (MOI), and growth conditions [28,29]. Conventional amplification in industrial fermenters (scaling to over 1 ton) typically requires phage seed stocks of 10^12^–10^13^ PFU with MOI values between 0.01 and 1.0, relying on bacterial cells in the exponential growth phase [30,31]. However, achieving and maintaining these quantities is challenging, especially for phages with low propagative strength or stability. For personalized phage therapy, “cognate phages”—phages specifically targeted to a patient’s infection—are particularly valuable [32,33]. Such applications typically require timely production of diverse phages in smaller quantities, with a single dose generally requiring around 10^9^ PFU [34,35,36], shifting the focus from bulk production to the rapid preparation of diverse, specific phages.

The agar plate method offers a practical alternative for phage propagation, harvesting phages from soft (top) agar overlays [37]. This method is widely used in phage laboratories due to its simplicity and is especially useful for phages that yield low quantities in liquid cultures or face propagation challenges due to host cell constraints [38,39]. Additionally, it provides clearer visualization of plaque formation, facilitating the assessment of phage activity and detecting contaminations. The yield from a single agar plate varies depending on the phage and bacterial strain but often provides enough material for multiple doses or seed stocks for larger-scale production [40,41]. Despite its advantages, the lack of standardized protocols for the agar plate method often results in inefficient use of resources, including inoculum and time. As demand for customized phage preparations grows, there is a need for standardized protocols enabling efficient high-mix, low-volume production to complement existing large-scale broth-based methods.

This study aims to standardize the agar plate method for the simultaneous production of various phages in basic microbiological facilities. Normalization between plaque size and PFU count was performed to ensure full coverage of standard petri dishes, employing a range of phages and bacterial species relevant to industrial sectors, including *Escherichia*, *Salmonella*, *Staphylococcus*, *Vibrio*, *Pseudomonas*, *Chryseobacterium*, *Erwinia*, and *Aeromonas*. Optimal production parameters were estimated based on relative inoculum volumes.

## 2. Results

### 2.1. Plaque Morphology

**Figure 1:**
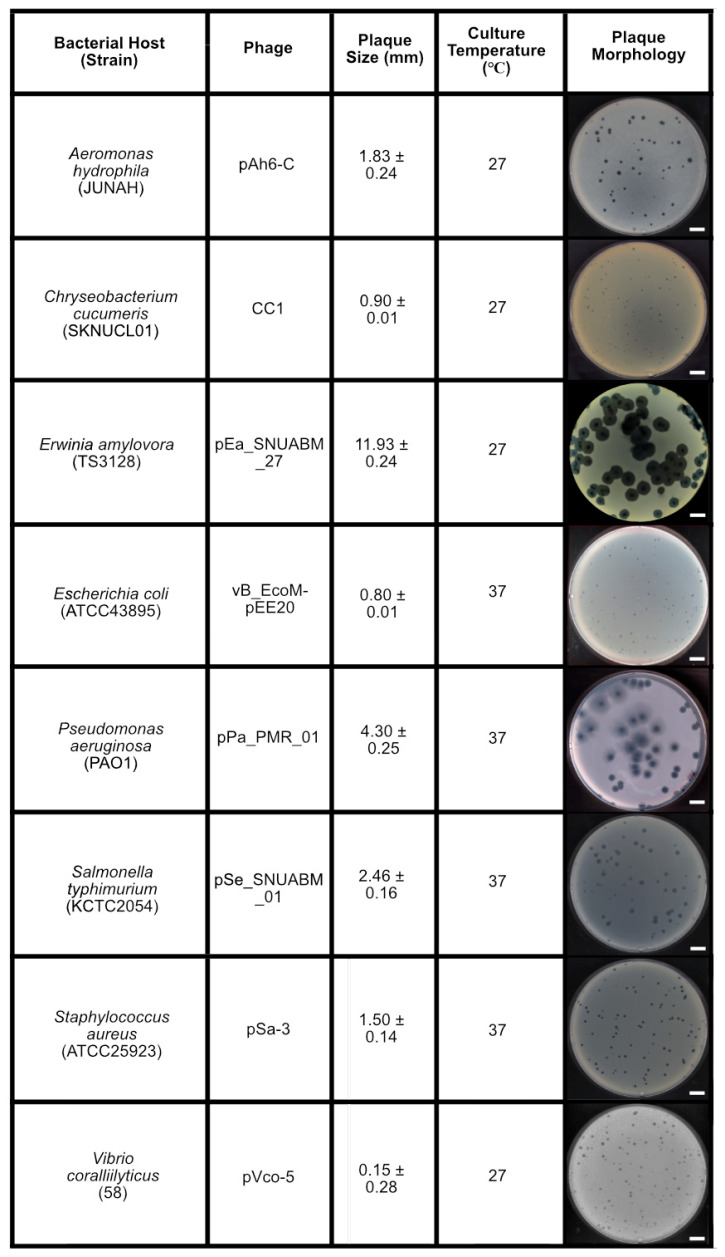
Microorganisms (bacteriophages and bacterial hosts) used in the study. Plaque assay was performed in 0.4% soft agar. Bar represents 10 mm.

We used previously isolated phages and the plaque sizes were observed on 0.4% soft agar, and the following observations were made: *Aeromonas* phage pAh6-C [42] formed plaques measuring 1.83 ± 0.24 mm in diameter; *Erwinia* phage pEa_SNUABM_27 [43] (hereafter, pEa_27), 11.93 ± 0.41 mm; *Escherichia* phage vB_EcoM-pEE20 (hereafter, pEE20), 0.80 ± 0.01 mm; *Salmonella* phage pSe_SNUABM_01 (hereafter, pSe_01), 2.46 ± 0.16 mm; *Staphylococcus* phage pSa-3 [44,45], 1.50 ± 0.14 mm; and *Vibrio* phage pVco-5 [46], 1.56 ± 0.28 mm (Figure 1). For the validation of the model, we isolated new phages: *Chryseobacterium* phage CC1, 0.90 ± 0.01 mm; *Pseudomonas* phage pPa_PMR_01 (hereafter, pPa_01), 4.30 ± 0.25 mm. Additionally, phage pEa_27 exhibited a smaller plaque size of 1.62 ± 0.08 mm when using 0.8% soft agar, which was specifically employed for validating the model.

### 2.2. Analysis of Inoculum and Plaque Area and Regression Model

**Figure 2:**
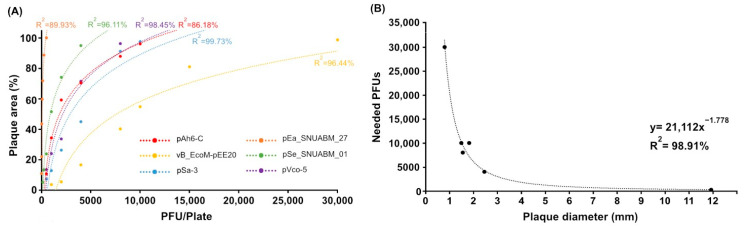
Regression model of phage plaque size and their relationship with plaque-forming units (PFUs). (**A**) The plot depicting the correlation between plaque area and the number of plated plaques for *Aeromonas* phage pAh6, *Erwinia* phage pEa_27, *Escherichia* phage vB_EcoM-pEE20, *Salmonella* phage pSe_01, *Vibrio* phage pVco-5, and *Staphylococcus* phage pSa-3. (**B**) Regression model demonstrating the relationship between plaque size and plaque number needed to achieve 100% coverage of plate. Equation = 21,112x^−1.778^ (R^2^ = 0.9891).

A comprehensive analysis was conducted using six previously isolated phages to elucidate the relationship between plaque formation and phage concentrations (Figure 2). For pAh6-C, the plaque coverage increased from 10.63% at 500 PFU to 34.22% at 1000 PFU, 59.21% at 2000 PFU, 70.34% at 4000 PFU, 87.90% at 8000 PFU, and finally 96.14% at 10,000 PFU. pEa_27 showed an increase in coverage from 10.78% at just 7.81 PFU to 22.05% at 15.62 PFU, 43.39% at 31.25 PFU, 59.64% at 62.5 PFU, 71.84% at 125 PFU, 88.79% at 250 PFU, and achieving complete coverage of 100% at 500 PFU. For vB_EcoM-pEE20, the coverage was 3.65% at 1000 PFU, 5.39% at 2000 PFU, 16.51% at 4000 PFU, 40.16% at 8000 PFU, 54.81% at 10,000 PFU, 81.02% at 15,000 PFU, and 98.72% at 30,000 PFU. The pSe_01’s plaque area expanded from 5.22% at 125 PFU to 13.32% at 250 PFU, 23.81% at 500 PFU, 51.46% at 1000 PFU, 74.06% at 2000 PFU, and 95.03% at 4000 PFU. pSa-3 demonstrated an increase in coverage from 7.30% at 500 PFU to 12.77% at 1000 PFU, 26.31% at 2000 PFU, 44.93% at 4000 PFU, 91.14% at 8000 PFU, and 97.57% at 10,000 PFU. Finally, pVco-5 displayed a coverage increase from 13.41% at 500 PFU to 23.94% at 1000 PFU, 33.57% at 2000 PFU, 71.47% at 4000 PFU, and 96.22% at 8000 PFU (Figure 2A). The R^2^ values were 86.18% for pAh6-C, 89.93% for pEa_27, 96.44% for vB_EcoM-pEE20, 96.11% for pSe_01, 99.73% for pSa-3, and 98.45% for pVco-5 (Figure 2A). The significance of the model was confirmed by F-values of 24.95 for pAh6-C, 44.67 for pEa_27, 135.47 for vB_EcoM-pEE20, 98.80 for pSe_01, 1485.46 for pSa-3, and 190.52 for pVco-5, supported by significant *p*-values (*p* < 0.05 for pAh6-C, *p* < 0.001 for pEa_27, vB_EcoM-pEE20, pSe_01, pSa-3, and pVco-5).

Based on the analysis derived from Figure 2B, the correlation between plaque size and the minimum-lawn cell clearing PFUs (MCPs) was examined. For instance, phage pAh6-C, which produces plaques with a diameter of 1.83 mm, required 10,000 PFUs to fully cover the plate, as evidenced in Figure 2A. pEa_27, which forms larger plaques of 11.93 mm, needed only 500 PFU for complete coverage. Phage vB_EcoM-pEE20, which produced the smallest plaques (0.80 mm), required 30,000 PFU for full coverage. Furthermore, phage pSe_01, with plaques of 2.46 mm, needed 4000 PFU; pSa-3, with 1.50 mm plaques, also required 10,000 PFU; and pVco-5, with plaques at 1.56 mm, required 8000 PFU. After determining these values, a regression model was developed using the collected data. The following equation describes the model:

$$y={21,112x}^{-1.778}$$

The Plaque Size Calculation (PSC) demonstrates a significant inverse correlation between plaque diameter (x) and the MCP (y) (Figure 2B). This relationship is evidenced by a robust R^2^ value of 98.91%. Furthermore, the regression model demonstrated exceptional statistical strength, with an F-value of 363.32 and a *p* < 0.001.

### 2.3. Validation of the Inoculum by PSC

**Figure 3:**
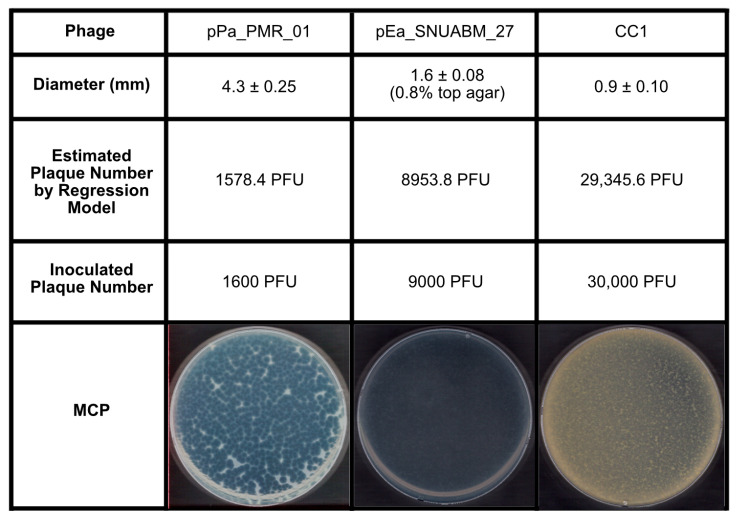
Validation of the regression model, plaque size calculation (PSC). The PSC was applied to predict MCPs for various phages, which were then used in plaque assays to evaluate the model’s accuracy.

Validation of the PSC involved a range of plaque sizes using both newly isolated phages, such as pPa_01 (4.30 mm) and CC1 (0.90 mm), as well as previously characterized phages, such as pEa_27 (Figure 3). For pEa_27, increasing the soft agar concentration from 0.4% to 0.8% reduced the plaque size from 11.93 ± 0.41 mm to 1.62 ± 0.08 mm, which was used for validation in this test. According to the PSC, the predicted MCPs were 1578.4 PFU for pPa_01, 8953.8 PFU for pEa_27 (at 0.8% soft agar), and 29,345.6 PFU for CC1. These values were validated by plating 1600 PFU, 9000 PFU, and 30,000 PFU, each achieving ~100% coverage and confirming the model’s accuracy.

### 2.4. Optimization of the Phage Inoculum

**Figure 4:**
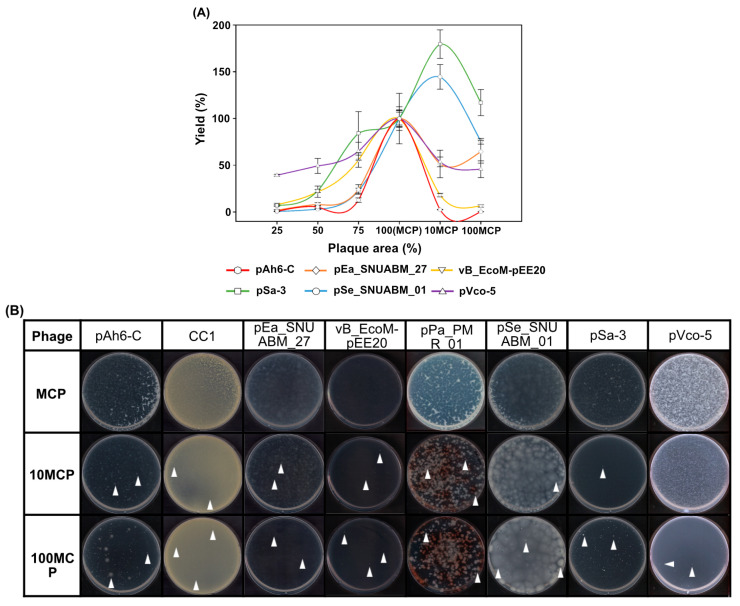
Phage propagation inoculum and yield analysis using the agar plate method. (**A**) Comparison of propagation yield based on the inoculation of MCPs. The inoculum varies from 1/4 to 100 times the MCPs. (**B**) Comparison of the emergence of bacteriophage resistance based on the inoculation of MCPs. Phage-resistant colonies are indicated by arrows as examples. The inoculum varies from 1 to 100 times the MCPs.

Harvest yields were assessed for phages pAh6-C, pEa_27, vB_EcoM-pEE20, pSe_01, pSa-3, and pVco-5 after plating at varying plaque area coverages, including 25%, 50%, 75%, 100% (MCP), 1000% (10× MCP), and 10,000% (100× MCP) (Figure 4A). All the phages reached their maximum productions when the inoculum reached optimal saturation levels between MCP and 10 MCP, ranging from 10^9^ to 10^11^ PFUs, followed by a decrease in production after saturation (Table 1). Interestingly, compared with MCP, a higher occurrence of bacteriophage-resistant strains was observed in most phages when the plaque area was seeded more than MCP (Figure 4B).

## 3. Discussion

With the rise of antimicrobial resistance (AMR), clinical trials using phages underscore the growing need for scalable phage production to treat resistant infections [47,48,49]. As phage therapy gains traction, improving production techniques becomes crucial. Most strategies follow a “pret-a-porter” model—broad-spectrum, standardized preparations of pre-prepared phage solutions aligning with regulatory requirements [50]. However, tailored protocols remain essential, as yields depend on each host and phage’s characteristics, such as the growth rate of the host bacteria, adsorption kinetics, and the burst size of phages [51,52,53]. In this study, we standardized the conventional agar plate method for phage propagation based on plaque diameter.

The therapeutic effect of phages is maximized through a “sur-mesure” approach, a precision medicine strategy that uses phages specifically infective to pathogenic bacteria [50]. This implies that in certain cases, such as when treating multidrug-resistant infections in hospital settings, on-site selection and propagation of tailor-made phage solutions may be required [54]. For hospitals dealing with untreatable infections, phage therapy can be most effectively implemented via this personalized approach, especially when pathogenic bacteria exhibit diverse types [55,56,57,58]. Moreover, since 10^9^ PFU of phages is often proposed as a single dose for treatment, large volumes of phage solution may not be necessary [34,59]. Our study demonstrated that the production of several doses could be easily achievable using the PSC model on agar plates (Table 1).

Numerous studies have described methods for phage propagation on agar plates, yet many lack a precise inoculation strategy [38,39,40,60,61]. Current methods vary, with two main approaches—wash-off and disruption—differing in the phage harvest step. In the wash-off method, plates with plaques are submerged in a buffer, and the supernatant containing diffused phages is collected [41]. The disruption method involves harvesting the overlaid soft agar, followed by mechanical shearing to release phages [39]. Both methods have limitations in determining inoculum doses, often relying on arbitrary rules of thumb, leading to inefficiencies. For example, small inoculum doses, such as 10^3^ PFU, may be insufficient for complete lysis in phages with small plaques, while high doses can result in lysis from without, diminishing proper phage propagation (Figure 2) [62]. However, when using a low-dose inoculum, as in the case of *Vibrio* phage pVa-21 with plaques smaller than 1 mm, this minimal inoculum was insufficient for complete bacterial lysis [63]. Conversely, using a high-dose inoculum with larger plaques, such as pEa_27 (11.9 mm), led to ineffective infection due to lysis from without (Figure 2). These challenges highlight the importance of precise inoculum selection.

To address this complexity, we focused on the common, essential feature of plaques rather than the distinct traits of individual phages. Complete lysis of the bacterial lawn on the agar plate is a reliable indicator of maximum virion production (Figure 2, 6) [39]. We then developed the PSC model to analyze the relationship between plaque size, initial PFU, and total phage production. While this study demonstrates the reliability and versatility of the PSC model, it is essential to note that the model was validated within the plaque size range of 3–12 mm. Plaque sizes were measured in conditions with 20–50 plaques per 90 mm Petri dish, and for phages with depolymerase activity, such as pEa_27, only the central plaques were measured, excluding the halo (Figure 1). Our model accurately predicted (R² = 98.91%) the number of PFUs required to cover a Petri dish (Figure 2B), verified with newly isolated phages, including *Pseudomonas* phage pPa_PMR_01 and *Chryseobacterium* phage CC1, which formed plaques of 4.3 mm and 0.9 mm, respectively, in 0.4% top agar. Additionally, phage pEa_27, which typically forms large plaques (11.9 mm), generated smaller plaques (1.6 mm) in 0.8% top agar, and an approximate MCP of 9000 PFU (model prediction: 8953 PFU) was sufficient to clear the bacterial lawn (Figure 3). Complete or near-complete clearing (lysis) of the bacterial lawn has been considered to peak phage production [41,64]. However, increasing the inoculum to 100 MCP led to reduced production and the evolution of phage-resistant bacterial mutants, as observed in previous study that indicated higher concentrations of phage led to a greater emergence of phage-insensitive mutants (Figure 4) [65]. This outcome, often referred to as “go farther and fare worse”, might occur when high phage populations induce lysis from without, reducing the actual phage infection and propagation [60,66]. Therefore, selecting an appropriate inoculum dose is crucial in optimizing phage production and minimizing resistance development [67].

**Figure 5:**
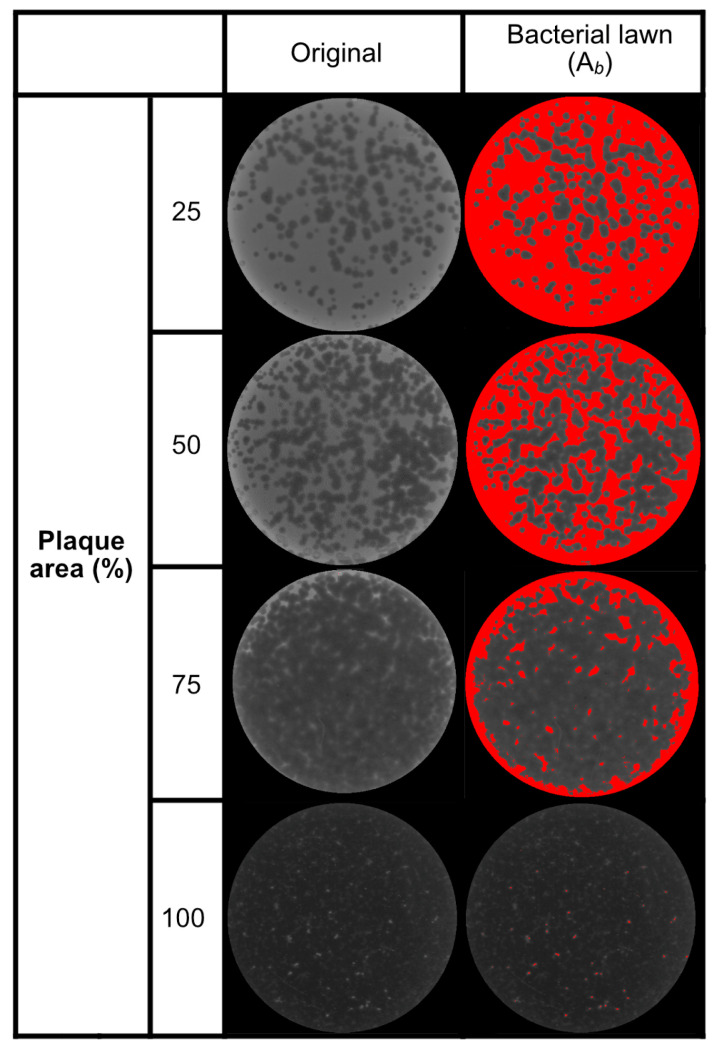
Schematic representation of plaque area measurement. The plaque area was calculated by subtracting the area occupied by the bacterial lawn (depicted in red) from the total area of the plate.

The PSC model, designed using standard 90 mm Petri dishes (63.6 cm² surface area), is easily scalable to larger formats, such as 150 mm plates, by adjusting the inoculum in proportion to the surface area (Figure 5). The utility of the agar plate method in amplifying phages has been validated across different phages and their bacterial hosts, which are major pathogens for industries [42,68,69,70]. The PSC model proposed here fills a gap in conventional methods, achieving significant improvements in production, with increases up to 2.2 Log PFU/mL in case of pAh6-C (Table 1). This would be particularly useful when working with bacteria such as *Mycobacterium* and *Nocardia* that grow in clumps [40,71]. The aggregation presents challenges for phage infection in liquid culture, resulting in compromised phage infection [45,53,72]. In these cases, liquid culture is not an ideal method; however, the agar plate method offers an efficient alternative for phage propagation, whether for laboratory experiments, industrial seed production, or rapid clinical application. Indeed, several phages have shown higher production when propagated in agar plate rather than broth culture [39,41].

It is also important to recognize that the bacterial infection by phages and plaque formation is a dynamic process and involves complex factors, such as superinfection exclusion and bacterial immunity [73,74,75,76,77,78,79]. These biological mechanisms can influence the size and number of plaques, potentially limiting the efficiency of phage propagation. Superinfection exclusion may restrict secondary phage infections, reducing plaque formation even at higher inoculum doses [74,75,76]. Additionally, bacterial immunity can lead to the emergence of phage-resistant strains during production, significantly decreasing overall population. [77,78,79]. A key limitation of this study is that the PSC model does not account for these host defense mechanisms, as it primarily focuses on plaque size and PFU. The model assumes uniform bacterial susceptibility, which may not always be the case in clinical or industrial settings, where bacterial populations are diverse.

Future studies should explore the effects of factors, such as superinfection exclusion, bacterial immunity, and propagation strain selection on phage production, and incorporate them into the PSC model to improve its accuracy. Selecting an optimal bacterial host is vital, as it greatly influences phage production. Overcoming both scientific and regulatory challenges will be essential to ensuring a reliable supply of phages for clinical use. Looking ahead, the PSC model holds promise for advancing small-scale phage production platforms, potentially integrating with bio-foundry systems to efficiently scale and streamline production.

## 4. Materials and Methods

### 4.1. Microorganisms and Culture Conditions

Bacterial strains and their corresponding phages were cultured according to protocols outlined in their respective reference reports, with temperature conditions specified in Figure 1. The host–phage pairs used in this study included *Aeromonas hydrophila* (JUNAH) and phage pAh6-C [42]; *Erwinia amylovora* (TS3128) and phage pEa_SNUABM_27 [43,68]; *Escherichia coli* (ATCC43895) and phage vB_EcoM-pEE20; *Salmonella enterica* (KCTC2054) and phage pSe_SNUABM_01; *Staphylococcus aureus* (ATCC25923) and phage pSa-3 [44,45]; *Vibrio coralliilyticus* (58) and phage pVco-5 [46,69]; *Chryseobacterium cucumeris* (SKNUCL01) [70] and phage CC1; *Pseudomonas aeruginosa* (PAO1) and phage pPa_PMR_01. All bacterial strains were cultured in tryptic soy broth (TSB; Becton, Dickinson and Company, Franklin Lakes, NJ, USA) and on tryptic soy agar (TSA; Becton, Dickinson and Company, Franklin Lakes, NJ, USA) and soft agar (TSB with 0.4% bacteriological agar, unless otherwise specified) for 18–24 h. For the culture of *Vibrio* species and its phage, the media were supplemented with 2% sodium chloride to accommodate the halophilic nature of the organism. The cultures were subsequently prepared for further analyses by inoculating 1% into TSB and cultivating the bacteria to the exponential phase (10^8^ CFU/mL).

### 4.2. Preparation of Bacteriophages

Propagation and the purification of the bacteriophages was performed as described in our previous study [80]. The lysate was precipitated using 10% (*w*/*v*) PEG and 0.5 M NaCl solution. A cesium chloride density gradient was utilized with ultracentrifugation (182,000× *g* for 3 h). The bacteriophage bands were isolated and dialyzed in a 10,000 MWCO membrane (Thermo Fisher Scientific Inc., Waltham, MA, USA) and the purified phage suspension (>10^10^ PFU/mL) was stored at 4 °C for further use. Phage titers in the sample were evaluated in triplicate using the plaque assay used as an indicator host strain.

### 4.3. Bacteriophage Plating, Plaque Area Analysis, and Harvest

All plating was performed using the conventional double-layer agar method [38]. Specifically, to establish the bottom agar layer, 10 mL of TSA was evenly poured into standard Petri dishes (90 mm in diameter). Plaque assays were performed using 0.4% or 0.8% soft agar. The soft agar mixtures were made by mixing 3 mL of 0.4% or 0.8% agar with 100 μL of a phage solution and 100 μL of bacterial host culture. The mixture was vigorously vortexed to ensure a thorough mixing of the bacteria and phages before being poured evenly over the bottom agar layer. Unless otherwise specified, assays were conducted with 0.4% soft agar and incubated for 18 h.

The plaque areas and bacterial lawns were quantified using ImageJ (ImageJ, Version 1.5, National Institutes of Health, Bethesda, MD, USA), and the ratio between them was calculated as follows:

$$Ratio\;of\;plaque(s)\;area=(A_{t}-A_{b})/A_{t}\times 100$$

where *A*_*t*_ is defined as the total area of the assay plate and *A*_*b*_ refers to the area occupied by the bacterial lawn. The area of the plaque was determined by deducting the area covered by the bacterial lawn (illustrated in red in Figure 5) from the total area of the plate.

To harvest the phages, 1 mL of SM buffer (50 mM Tris [pH 7.5], 100 mM NaCl, and 10 mM MgSO_4_) was added to scrape off the soft agar. The collected soft agar was then disrupted by repetitive pipetting to break down the soft agar matrix. The mixture was subjected to centrifugation at 12,000× *g*, followed by filtration through a 0.45 µm Millipore filter prior to titration. Previously isolated phages, including pAh6-C, pEa_27, pSe_01, vB_EcoM-pEE20, pSa-3, and pVco-5, were used to construct a regression model that establishes a correlation between plaque size and the number of PFUs for full plates. To assess the model’s validity, newly isolated phages CC1 and pPa_01 were used. Furthermore, to assess the model’s versatility, the soft agar concentration was varied from 0.4% to 0.8%, which altered the plaque sizes of the phage pEa_27 from 11.93 mm to 1.62 mm.

### 4.4. Statistical Analysis

Statistical analysis was performed using Minitab(Version 22, Minitab, LLC, State College, PA, USA). The analysis of plaque area and inoculum, and the regression model, was conducted using the Fitted Line Plot. A *p*-value < 0.05 was considered significant.

## 5. Conclusions

This study developed the PSC model, linking plaque size, inoculum concentration, and harvest yield. Precise inoculum density control was crucial for optimizing phage propagation, with specific PFU requirements for full plaque coverage. This study identified an inoculum density threshold that maximizes production and minimizes resistant bacteria. Validation across various bacteriophages confirmed the model’s effectiveness and adaptability, highlighting its potential as a standard tool in clinical phage production, especially for handling diverse phages in small quantities and selecting suitable ones through screening.

## Figures and Tables

**Table 1:** Total phage production in a Petri dish (90 mm) based on plaque area.

	Plaque Area (%)					
	25	50	75	100 (=MCP)	10 MCP	100 MCP
pAh6-C	5.17 ± 0.38 × 10^9^ PFU/mL	4.50 ± 0.11 × 10^10^ PFU/mL	1.05 ± 0.20 × 10^10^ PFU/mL	8.33 ± 0.22 × 10^11^ PFU/mL	1.97 ± 0.26 × 10^10^ PFU/mL	1.43 ± 0.32 × 10^9^ PFU/mL
pEa_27	4.33 ± 1.88 × 10^7^ PFU/mL	2.03 ± 0.46 × 10^8^ PFU/mL	6.10 ± 0.12 × 10^8^ PFU/mL	2.53 ± 0.23 × 10^9^ PFU/mL	1.30 ± 0.37 × 10^9^ PFU/mL	1.63 ± 0.30 × 10^9^ PFU/mL
vB_EcoM-pEE20	1.02 ± 0.17 × 10^9^ PFU/mL	2.80 ± 0.77 × 10^9^ PFU/mL	7.20 ± 0.98 × 10^9^ PFU/mL	1.29 ± 0.16 × 10^10^ PFU/mL	2.33 ± 0.24 × 10^9^ PFU/mL	8.23 ± 0.16 × 10^8^ PFU/mL
pSe_01	5.30 ± 0.72 × 10^8^ PFU/mL	1.72 ± 0.12 × 10^9^ PFU/mL	6.40 ± 0.17 × 10^9^ PFU/mL	7.60 ± 0.64 × 10^9^ PFU/mL	1.37 ± 0.11 × 10^10^ PFU/mL	8.90 ± 0.10 × 10^9^ PFU/mL
pSa-3	1.07 ± 0.23 × 10^8^ PFU/mL	7.40 ± 0.73 × 10^8^ PFU/mL	5.86 ± 0.67 × 10^9^ PFU/mL	2.58 ± 0.17 × 10^10^ PFU/mL	3.73 ± 0.33 × 10^10^ PFU/mL	1.95 ± 0.77 × 10^10^ PFU/mL
pVco-5	1.83 ± 0.47 × 10^9^ PFU/mL	2.30 ± 0.37 × 10^9^ PFU/mL	3.03 ± 0.45 × 10^9^ PFU/mL	4.67 ± 0.41 × 10^9^ PFU/mL	2.50 ± 0.24 × 10^9^ PFU/mL	2.13 ± 0.41 × 10^9^ PFU/mL

## Data Availability

Data are contained within the article.
